# Immunosenescence and Allergy: Molecular and Cellular Links Between Inflammaging, Neuro-Immune Aging, and Response to Biologic Therapies

**DOI:** 10.3390/ijms27031206

**Published:** 2026-01-25

**Authors:** Ernesto Aitella, Gianluca Azzellino, Barbara Antonella Cammisuli, Carmen De Benedictis, Domenica Di Mattia, Ciro Romano, Lia Ginaldi, Massimo De Martinis

**Affiliations:** 1 Department of Life, Health and Environmental Sciences, University of L’Aquila, 67100 L’Aquila, Italy; gianluca.azzellino@graduate.univaq.it (G.A.); barbaraantonella.cammisuli@graduate.univaq.it (B.A.C.); carmen.debenedictis@graduate.univaq.it (C.D.B.); domenica.dimattia@student.univaq.it (D.D.M.); lia.ginaldi@univaq.it (L.G.); 2 Allergy and Clinical Immunology Unit, Center for the Diagnosis and Treatment of Osteoporosis, AUSL 04 Teramo, 64100 Teramo, Italy; 3 Complex Operational Unit, Adriatic District Area, AUSL 04 Teramo, 64100 Teramo, Italy; 4 Clinical Immunology Outpatient Clinic, Division of Internal Medicine, Department of Advanced Medical and Surgical Sciences, “Luigi Vanvitelli” University of Campania, 80131 Naples, Italy; ciro.romano@unicampania.it; 5 Long-Term Care Unit, “Maria SS. dello Splendore” Hospital, AUSL 04 Teramo, 64021 Giulianova, Italy; 6 UniCamillus-Saint Camillus International University of Health Sciences, 00131 Rome, Italy

**Keywords:** immunosenescence, allergic diseases, inflammaging, neuro-immune aging, type 2 inflammation, biologic therapies, elderly

## Abstract

With the global increase in population aging, allergic diseases in older adults are becoming an increasingly relevant clinical and public health challenge. Age-related molecular and cellular alterations significantly affect the pathophysiology, clinical manifestations, diagnosis, and management of major allergic diseases in the elderly. This review focuses on immunosenescence in major allergic conditions, including asthma, chronic urticaria and angioedema, dermatitis, food and drug allergies, and hymenoptera venom hypersensitivity. Particular emphasis is placed on molecular mechanisms underlying immune aging, such as inflammaging, dysregulation of innate and adaptive immune responses, epithelial barrier dysfunction, microbiota alterations, neuro-immune interactions, and age-related comorbidities. Sex-related differences in immune responses are also addressed, together with current diagnostic and therapeutic strategies, including the opportunities and limitations of biologic therapies in aging populations. Despite growing interest in this field, a major limitation remains the paucity of studies specifically targeting geriatric populations, underscoring the need for age- and sex-specific research and dedicated clinical trials. A personalized approach integrating frailty assessment and immune profiling is essential to optimize the management of allergic diseases in older adults.

## 1. Introduction

By 2050, the global population of adults aged 65 years and older is projected to exceed 1.6 billion, making age-related diseases and conditions an increasingly pressing public health concern [1]. In this context, allergic diseases in older adults represent an emerging and still underestimated clinical challenge, with a reported prevalence of approximately 5–10% in the elderly population [2]. An in-depth characterization of the molecular and cellular mechanisms underlying immune remodeling with aging is essential to define immunosenescence and its contribution to age-related diseases, including allergic disorders. The study of age-related immunological processes is therefore critical for accurate diagnosis, appropriate therapeutic strategies, and the advancement of precision medicine tailored to older individuals.

Two hallmark phenomena of aging are cellular senescence and inflammaging. The former encompasses permanent cell cycle arrest, telomere shortening, resistance to apoptosis, and the acquisition of a senescence-associated secretory phenotype (SASP) [3], while the latter refers to a state of chronic, low-grade, sterile inflammation that progressively increases with age and is commonly associated with elevated inflammatory markers [4]. Clarifying how these mechanisms influence the pathophysiology, clinical expression, and management of allergic diseases, long regarded as conditions primarily affecting childhood and adulthood, is essential to optimize care in the aging population. However, the limited availability of age-specific data represents a significant constraint, since results derived from younger or mixed-age populations are not directly transferable to older adults due to immunosenescence, multimorbidity, polypharmacy, and frailty.

The aim of this review is to critically examine the impact of immunosenescence and inflammaging on the molecular, pathophysiological, and clinical features of major allergic diseases, including asthma, urticaria, angioedema, and food, drug, and hymenoptera venom hypersensitivity.

## 2. Molecular Mechanisms of Immunosenescence

The immune system undergoes functional changes with aging, driven by alterations in both innate and adaptive immune responses [5] and further influenced by the multiple comorbidities common in older adults. The endocrine system also experiences an age-related decline, marked by a progressive reduction in the activity of major glands [6]. In addition, older individuals frequently suffer from chronic conditions that progressively impair organ function, including chronic kidney disease, chronic obstructive pulmonary disease (COPD), liver dysfunction, heart disease, cognitive decline, depression, vitamin deficiencies, and intestinal dysbiosis, the latter often exacerbated by extensive antibiotic use. Consequently, elderly patients are typically treated with multiple medications, which places additional strain on excretory organs. Environmental factors such as long-term smoking, exposure to pollution, and physical and psychological stressors further contribute to pathological aging [7,8].

The following section explores age-related changes in immune system responses.

### 2.1. Decline in Hematopoietic Stem Cells

The sequential loss and shortening of telomeric DNA with advancing age lead to increased apoptosis. In addition to telomere attrition, acquired defects in genomic and mitochondrial DNA further contribute to the age-related decline in hematopoietic stem cell function. Overall, the proliferative capacity of hematopoietic stem cells in older individuals is estimated to be two to four times lower than in younger adults [9].

Pro-B cell production declines markedly with age, resulting in a reduced output of B cells from the bone marrow, whereas T cell precursors appear to be comparatively less affected. In contrast, erythroid and myeloid progenitor compartments are relatively preserved, and available evidence indicates an age-associated skewing of hematopoiesis toward the myeloid lineage, reflecting a preferential commitment of aging hematopoietic stem cells to myelopoiesis [10,11,12,13].

### 2.2. Innate Immunity

The innate immune system comprises epithelial barriers, macrophages, neutrophils, natural killer (NK) cells, natural killer T cells, dendritic cells, and complement proteins. Although some innate immune mechanisms become impaired with aging, others appear to be upregulated, ultimately contributing to a greater tendency toward chronic inflammatory states. Therefore, immunosenescence of the innate immune system is more accurately described as a state of immune dysregulation characterized by chronic, low-grade sterile inflammation [14].

More specifically, macrophage function [15,16] declines overall due to both a reduction in precursor cell numbers and diminished TNF-α production. Neutrophil counts remain largely unchanged in the elderly, but their phagocytic capacity is reduced. Eosinophils exhibit impaired degranulation and diminished superoxide production [17].

Regarding NK cells, the CD56^dim^ (low CD56-expressing) subset increases with age, whereas the CD56^bright^ (high CD56-expressing) subset decreases [18]. Total NK cell numbers tend to rise, and their cytolytic function appears to be largely preserved, while overall IFN-γ production is only moderately compromised [14]. Indeed, the predominance of the immunoregulatory subset over the more cytotoxic population functionally implies a diminished capacity for antiviral defense and tumor immunosurveillance; this shift enhances pro-inflammatory cytokine production, thereby sustaining chronic inflammation and promoting inflammaging [19].

### 2.3. Adaptive Immunity

Adaptive immunity is mediated by T and B lymphocytes, which coordinate cellular and humoral responses, respectively. The most striking age-related alteration is thymic involution, resulting in a sharp decline in the number of naïve T cells produced and exported from the thymus. This decline becomes progressively more pronounced across the 40–54, 55–69, and 70–90 age groups [20].

Overall, T cell function decreases with aging, accompanied by an increase in CD8^+^ cells and a reduction in CD4^+^ cells. IL-2 secretion capacity is generally diminished with pathological aging, whereas IL-6 levels are increased, despite their potential bidirectional, concentration-dependent effects on immune regulation [21]. The number of regulatory T cells (Tregs) declines, which may contribute to the increased prevalence of autoimmune diseases and cancer in older adults [22]. Another hallmark of immunological senescence is the expansion of CD28^−^CD57^+^ T lymphocytes, characterized by low proliferative capacity, defective costimulatory signaling, and a pro-inflammatory contribution [23].

### 2.4. Humoral Immunity and B Cells

The number of B cell precursors in the bone marrow (pre-B cells), as well as peripheral B cells, decreases with age. The levels of specific antibodies (i.e., those generated by encountering antigens through infections or vaccinations) decrease with age. Some studies have shown that, similarly to T cells, the diversity of the B cell repertoire declines with age. However, while naïve B and T cell compartments are particularly affected by immunosenescence, antigen-experienced memory B and T cell subsets generated earlier in life tend to be relatively preserved or maintained, reflecting an age-dependent and dynamic reconfiguration of adaptive immune memory rather than a uniform decline across all lymphocyte populations [24,25,26].

### 2.5. Allergy-Specific Features: From Mast Cells to Microbiota

Aging profoundly remodels the innate immune system, generating a pro-inflammatory and dysfunctional microenvironment that shapes allergic phenotypes in older adults. Mast cells notably shift toward a phenotype characterized by enhanced basal cytokine and reactive oxygen species (ROS) release [27,28]. By contrast, eosinophils display impaired chemotaxis, reduced responsiveness to IL-5 and GM-CSF, and shortened survival, contributing to the chronicity of allergic conditions and reduced therapeutic responsiveness [29]. Dendritic cells are similarly affected, with diminished antigen presentation, attenuated type I interferon production, and altered Toll-like receptor signaling [30,31].

A key molecular driver of these alterations is the senescence-associated secretory phenotype (SASP), characterized by sustained release of IL-6, IL-8, IL-1β, TNF-α, chemokines, matrix metalloproteinases, alarmins, and ROS [32,33,34]. SASP disrupts epithelial integrity, fuels leukocyte recruitment, and amplifies Th2, Th17, and innate lymphoid cell responses, thereby linking immunosenescence, inflammaging, and persistent allergic inflammation [35,36]. Mitochondrial dysfunction and age-related oxidative stress further intensify this process by increasing ROS burden, reducing ATP production, activating the NLRP3 inflammasome, enhancing IL-1β and IL-18 secretion, and impairing epithelial repair [36,37].

In addition, aging-associated shifts in gut, skin, and airway microbiota, particularly with the loss of short-chain fatty acid-producing commensals, compromise barrier function and immune tolerance, facilitating allergen penetration and promoting chronic inflammation [38,39].

### 2.6. Neurogenic Inflammation and Neuro-Immune Aging

Due to the mast cell’s anatomical and pathophysiological localization [40] at the crossroads between hypersensitivity and neurogenic inflammation, neuro-immune aging adds another regulatory layer: altered neuropeptide release (e.g., substance P, calcitonin gene-related peptide-CGRP), reduced sensory nerve density, and dysregulated autonomic signaling enhance neurogenic inflammation, contributing to chronic pruritus, neurodermatitis, and non-IgE-mediated hypersensitivity [41,42].

Collectively, these mechanisms establish a self-perpetuating feedback loop in which immunosenescence drives inflammaging, thereby amplifying allergic inflammation, oxidative stress, and tissue damage in a bidirectional, age-dependent cycle. In particular, neuro-immune remodeling is driven by cumulative molecular and cellular dysfunction that alters inflammatory homeostasis and immune surveillance. Innate immune cells display elevated basal activation associated with dysregulated pattern recognition receptor signaling and impaired effector responses to acute stimuli [43].

Within the central nervous system, systemic immune alterations converge with intrinsic glial aging programs to drive maladaptive neuro-immune activation. Microglia undergo priming and dystrophic remodeling characterized by persistent activation of pro-inflammatory signaling pathways, increased basal production of cytokines and chemokines, impaired phagocytosis, defective clearance of cellular debris, and dysregulated inflammasome activity [44]. On the other side, astrocytes adopt hyperreactive phenotypes marked by metabolic dysregulation, mitochondrial fragmentation, and decreased expression of synaptogenic factors, leading to compromised synaptic maintenance [45].

The sustained release of inflammatory mediators contributes to the disruption of blood–brain barrier integrity through altered endothelial signaling and junctional protein dysfunction, facilitating the infiltration of peripheral immune cells into the brain parenchyma [46]. This process amplifies local inflammatory signaling and further perturbs neuron–glia communication. As a consequence, neurons experience progressive bioenergetic failure, synaptic dysfunction, and increased susceptibility to cytokine- and oxidative stress-mediated damage. Together, the chronic molecular dysregulation of systemic immunity, glial activation, and neuronal stress responses establishes a feed-forward loop of inflammation and cellular dysfunction. This environment lowers the threshold for neurodegenerative vulnerability and contributes to the initiation and progression of age-related neurodegenerative diseases, including Alzheimer’s disease, Parkinson’s disease, and multiple sclerosis [47,48,49].

### 2.7. Immunosenescence and Allergy: An Integrated View

The main molecular mechanisms underlying immunosenescence are summarized in Table 1 and discussed in detail below.

With aging, coordinated defects across adaptive, innate, and tissue-level regulatory pathways converge toward chronic low-grade inflammation, reduced tolerance, and heterogeneous allergic phenotypes in older adults. Adaptive immune remodeling is dominated by T cell senescence, with accumulation of CD28^−^CD57^+^ subsets, showing reduced IL-2 production, altered cytokine profiles, an imbalance in Th1/Th2/Th17, and atypical allergic manifestations. In parallel, B cell aging is characterized by the expansion of exhausted memory compartments and impaired germinal center reactions, leading to reduced class-switch recombination and affinity maturation, and consequently lower functional antibody affinity, including IgE and IgG, which may shape clinical heterogeneity [24,26].

Innate and effector alterations further modulate disease expression. Mast cells exhibit FcεRI signaling changes and heightened oxidative stress, promoting basal mediator release and atypical presentations of urticaria, chronic eczema, and neurogenic inflammation. Eosinophils show reduced responsiveness to IL-5 receptor and GM-CSF signals, with impaired recruitment and persistence, potentially contributing to modified asthma phenotypes and variable responses to eosinophil-targeting biologics in the elderly [27,28,29].

Cellular senescence amplifies immune dysregulation through SASP-driven release of IL-6, IL-8, IL-1β, TNF-α, matrix metalloproteinases, and alarmins, which disrupt barrier integrity and perpetuate sterile inflammation. Mitochondrial dysfunction and ROS excess reinforce these effects via NLRP3 inflammasome activation and increased IL-1β/IL-18 production, impairing tissue repair and increasing fragility during exacerbations [35,36]. Dysbiosis of gut and skin microbiota, linked to altered short-chain fatty acid production and Toll-like receptor signaling, further compromises epithelial defenses and tolerance, facilitating allergen penetration and chronic sensitization [31,52]. Finally, neuro-immune aging, as well as substance P and CGRP dysregulation, enhances neurogenic inflammation and contributes to chronic pruritus, neurodermatitis, and non-IgE-mediated hypersensitivity, often challenging to manage in older adults [42,54].

**Figure 1:**
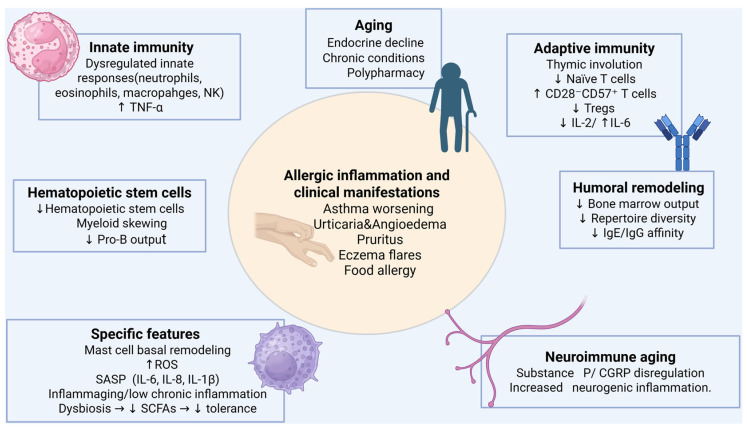
Integrated model of age-related allergic susceptibility. Aging is associated with endocrine decline, chronic comorbidities, polypharmacy, and progressive immune remodeling, affecting both innate and adaptive compartments. Innate immunity shows dysregulated responses of neutrophils, eosinophils, macrophages, and natural killer (NK) cells, with increased tumor necrosis factor-α (TNF-α) production. Adaptive immunity is characterized by thymic involution, reduced naïve T cell output, expansion of senescent CD28^−^CD57^+^ T cells, decreased regulatory T cells (Tregs), and an imbalance of cytokine signaling (↓ interleukin-2 (IL-2)/↑ interleukin-6 (IL-6)). Hematopoietic stem cell dysfunction leads to reduced stem cell pools, myeloid skewing, and decreased pro-B cell output, contributing to humoral remodeling with reduced repertoire diversity and decreased immunoglobulin E/immunoglobulin G (IgE/IgG) affinity. Additional age-related features include mast cell basal remodeling, oxidative stress with increased reactive oxygen species (ROS), senescence-associated secretory phenotype (SASP; IL-6, IL-8, and IL-1β), chronic low-grade inflammation (“inflammaging”), and dysbiosis with reduced short-chain fatty acids (SCFAs) and immune tolerance. Neuro-immune aging, driven by dysregulation of Substance P and calcitonin gene-related peptide (CGRP), further promotes neurogenic inflammation. These mechanisms sustain allergic inflammation and contribute to clinical manifestations such as asthma worsening, urticaria/angioedema, pruritus, eczema, and food allergy in older adults. Created in BioRender.com. Ginaldi, L. (2026) https://BioRender.com/j110ft7 (accessed on 20 January 2026).

Building on these molecular and cellular mechanisms, the following sections will discuss the clinical implications for allergy in older adults (Figure 1), including disease presentation, diagnostic challenges, and management strategies.

## 3. Lung Aging and Allergic Asthma

Asthma has traditionally been considered a disease affecting primarily children and young adults. However, with population aging, its prevalence among individuals over 65 years of age has increased, reaching an estimated 6–10%. Mortality among elderly patients with asthma is significantly higher [11]. In a large U.S. database study conducted between 2006 and 2008, older adults with asthma had a fivefold increased risk of all-cause mortality compared with younger adults, even after adjusting for comorbidities [56]. Moreover, an age-dependent relative risk of mortality exceeding sevenfold was observed among individuals aged 90–94 years in more recent years [57].

Asthma in older adults may represent either childhood-onset disease persisting into advanced age or asthma that develops de novo later in life. Classic symptoms—such as wheezing, coughing, and dyspnea—are often absent or atypical, leading to diagnostic confusion with conditions such as heart failure or chronic obstructive pulmonary disease (COPD), and resulting in delayed or missed diagnoses. Diagnostic tools such as spirometry and bronchodilator testing are frequently underutilized, partly due to suboptimal test performance in this population [58,59].

It is likely that a substantial proportion of older individuals diagnosed with COPD actually have long-standing, undertreated asthma that has progressed to an irreversible stage. Increased awareness and recognition of asthma in the elderly could reduce the number of misclassified COPD cases.

Aging is associated with structural and functional deterioration of the lung parenchyma, including reduced elasticity (due to loss of elastic fibers and alterations in collagen), changes in extracellular matrix composition, impaired mucociliary clearance, and senescence of lung stem cells, characterized by reduced proliferation and increased apoptosis [60]. These structural changes are compounded by age-related immune dysregulation, which promotes excessive and inappropriate production of pro-inflammatory cytokines such as IL-1β, IL-6, TNF-α, and senescence-associated secretory phenotype (SASP) factors [61]. This chronic low-grade inflammation impairs tissue repair and regeneration. Immunosenescence and inflammaging contribute to the late-onset of asthma in adults and older individuals and may be further exacerbated by external stimuli, including fungi, viruses, environmental pollutants, intestinal dysbiosis, smoking, and danger-associated molecular patterns [62].

Although allergic asthma is typically mediated by type 2 inflammation, in elderly individuals it frequently involves additional non-Th2 inflammatory pathways [63]. Older patients often exhibit increased sputum neutrophilia and elevated levels of TNF-α [64]. The coexistence of Th2-driven and non-Th2 inflammation results in a complex clinical phenotype that is more challenging to manage with current therapies [19]. Older adults may also show a diminished response to anti-IgE biologic agents. Nevertheless, accumulating evidence indicates that patients with higher IgE levels and eosinophil counts continue to derive clinical benefit from biologic therapy, which therefore should not be withheld solely on the basis of age [22]. A retrospective study evaluating the safety of four biologics (omalizumab, mepolizumab, reslizumab, and benralizumab) in patients over 70 years of age demonstrated a safety profile comparable to that observed in younger populations [65]. Additional retrospective data support similar therapeutic responses between younger and older adults [66]. Newer agents, such as tezepelumab, have been less extensively studied in elderly patients.

Therapeutic strategies are often based on guidelines developed for younger populations, with limited consideration of age-related immune and clinical heterogeneity. With regard to first-line inhaled therapies, many geriatric patients experience difficulty mastering proper inhalation technique, which is essential for treatment efficacy. This difficulty correlates with the degree of cognitive impairment [67], which is more prevalent in older adults. Adequate patient education and the use of appropriate inhalation aids are therefore crucial. Even in patients who learn the correct technique, low inspiratory flow may limit the effectiveness of certain devices [68]. Peak flow measurement can help assess inhalation capacity and guide device selection.

Inhaled corticosteroids and bronchodilators are generally well tolerated in older adults, even in the presence of multiple comorbidities. Nevertheless, careful monitoring is required to prevent adverse effects such as oral candidiasis and hyperglycemia. In patients requiring oral glucocorticoids, both dosage and duration should be carefully controlled to minimize serious side effects [69]. Another drug with bronchodilator properties that has been used in obstructive lung diseases is theophylline [70,71]; however, it is contraindicated in most elderly patients due to its variable metabolism and numerous drug interactions. Chronic theophylline toxicity can result in severe and potentially fatal outcomes [72].

## 4. Allergic Rhinitis in the Elderly

Allergic rhinitis is characterized by inflammation of the nasal mucosa and typically presents with sneezing, rhinorrhea, and nasal obstruction. These symptoms are often accompanied by itching of the eyes, nose, and palate, postnasal drip, and cough [73]. With increasing life expectancy, this condition is becoming more common in the elderly [74]. Symptoms in older adults are frequently misattributed to other forms of rhinitis, including vasomotor, infectious, or drug-induced rhinitis. Despite age-related changes in immune function, specific IgE production may persist into advanced age, allowing the allergic response to continue. Furthermore, nasal mucosal atrophy, reduced mucociliary clearance, and structural alterations can modify the clinical presentation of allergic rhinitis [75].

Diagnosis and management in older adults are often complicated by comorbidities. Nearly 50% of individuals over 75 years old have three or more chronic conditions and take three or more medications [76]. A detailed medical history, including an assessment of environmental and occupational exposures, is essential for identifying allergens or triggers that exacerbate symptoms. The primary therapeutic approach remains allergen avoidance. For example, in cases of dust mite or mold sensitivity, measures such as carpet removal, pet avoidance, and frequent washing of bedding are particularly important for elderly individuals who spend most of their time indoors [77].

Daily nasal irrigation with large volumes (approximately 150 mL) of hypertonic saline has shown moderate benefit compared with placebo [78]. Mucolytics may also provide symptomatic relief. Regarding pharmacologic therapy, second-generation antihistamines are considered safe in older adults because they lack significant anticholinergic and alpha-adrenergic effects [79]. In contrast, first-generation antihistamines should be avoided due to their central nervous system adverse effects and extensive drug interaction profiles. Among topical agents, azelastine has demonstrated good tolerability in adults [80] and increased effectiveness when combined with intranasal corticosteroids [81]; it also appears to be safe in elderly populations [82].

Systemic decongestants containing alpha-adrenergic agonists should generally be avoided in older adults, particularly those with coronary artery disease, diabetes, hypertension, hyperthyroidism, narrow-angle glaucoma, or bladder outlet obstruction [83]. Leukotriene receptor antagonists, such as montelukast, may reduce inflammation and alleviate congestion, sneezing, and rhinorrhea. Although relatively weak as monotherapy, they are often used in combination with antihistamines and appear to be well tolerated in geriatric patients [84]. Intranasal sodium cromoglycate may be effective in reducing symptoms in refractory cases. By inhibiting mast cell degranulation, it prevents the release of inflammatory mediators and may represent a suitable option for elderly patients who cannot tolerate antihistamines or decongestants, or for those taking multiple medications, given its minimal potential for drug interactions [85].

Immunotherapy is considered a final therapeutic option for patients with persistent moderate-to-severe symptoms despite medical treatment. Although few studies have evaluated its efficacy in older adults, available evidence is generally encouraging [86,87].

Surgical intervention may also be considered in selected geriatric patients. Paranasal sinus surgery has been shown to be safe and effective in improving quality of life in the elderly [88]. However, functional status must be carefully assessed to determine surgical suitability.

## 5. Immunosenescence and Skin Diseases: Urticaria, Angioedema, and Dermatitis

With aging, the incidence of chronic urticaria, angioedema, and both atopic and contact dermatitis increases significantly [89,90]. In older adults, the clinical presentation is often atypical, with less pronounced erythema and reduced or underreported pruritus, sometimes due to diminished sensory perception or neurological comorbidities. These manifestations may resemble non-allergic conditions such as xerosis, senile eczema, or prurigo secondary to scratching.

This atypical presentation can be explained by age-related degenerative changes of the skin. Aging leads to epidermal thinning, reduced dermal collagen and elastin, and a decline in sebaceous gland activity, resulting in decreased elasticity, tone, and hydration. These structural alterations impair barrier function and modify inflammatory responses, reducing local vasodilation and rendering erythema less clinically evident [91]. Consequently, diagnosis may be delayed, and the persistent lack of age-focused research represents a missed opportunity to advance precision medicine in dermatoallergology, ultimately postponing appropriate treatment.

Aging induces both structural and immunological alterations in the skin. Epidermal thinning, reduced hydration, microbiome changes, and lipid barrier disorganization are commonly observed [27,92]. From an immunological perspective, both the number and functional capacity of Langerhans cells decline, compromising antigen presentation and cutaneous immune surveillance. In addition, chronic hyperactivation of mast cells and basophils may occur, promoting spontaneous release of inflammatory mediators even without IgE involvement, contributing to spontaneous urticaria or irritant dermatitis. Elderly skin also exhibits reduced recruitment of effector T cells, altered chemokine expression, and decreased antimicrobial peptide production, increasing susceptibility to recurrent infections [93]. These mechanisms are further sustained by systemic “inflammaging,” characterized by chronically elevated levels of IL-6, TNF-α, and IL-1β.

### 5.1. Chronic Urticaria and Angioedema

In older adults with chronic urticaria, mast cells display an increased tendency toward spontaneous histamine release, coupled with reduced inhibitory regulation. Immunosenescence contributes to this dysregulation through altered innate immune homeostasis and heightened baseline pro-inflammatory activity [94,95]. Autoimmune mechanisms are common: approximately 40–45% of patients develop IgG autoantibodies against the high-affinity IgE receptor (FcεRI) or against IgE itself, promoting mast cell degranulation in the absence of allergens [96,97]. Alternatively, chronic urticaria has also been associated with gastrointestinal complaints, particularly gastroesophageal reflux disease, potentially driven by neurogenic dysregulation and viscero-somatic cross-sensitization [98].

Bradykinin-mediated angioedema represents a distinct entity in which bradykinin rather than histamine is the primary mediator. In elderly individuals, it may result from late-onset somatic mutations or acquired C1 esterase inhibitor (C1-INH) deficiency, often associated with lymphomas, monoclonal gammopathies, or subclinical autoimmune disorders [99]. Immunosenescence favors these conditions by promoting autoimmunity, complement dysregulation, and impaired vascular inflammatory control. Idiopathic angioedema may be misdiagnosed and confused with ACE inhibitor-induced angioedema, systemic amyloidosis, or lymphoproliferative disease [100]. In cases of angioedema without urticaria, a bradykinin-mediated mechanism should be suspected [101]. In older adults, acquired C1-INH deficiency may accompany chronic lymphocytic leukemia, non-Hodgkin lymphoma, Waldenström macroglobulinemia, or AL amyloidosis [102]. These disorders promote C1-INH consumption or neutralization via autoantibodies, leading to complement pathway hyperactivation, bradykinin accumulation, and tissue edema. Diagnostic evaluation should therefore include functional and antigenic C1-INH levels, as well as C4 and C1q measurements, to identify underlying hematologic causes. Delayed diagnosis is common and may worsen outcomes, particularly in patients unresponsive to antihistamines or with recurrent angioedema without urticaria [89,103].

### 5.2. Atopic and Contact Dermatitis

Atopic dermatitis may first appear later in life as late-onset atopic dermatitis (LOAD), a phenotype with distinct clinical and immunologic features compared with childhood disease. LOAD is often intrinsic, meaning non-IgE-mediated, with lower rates of systemic allergic sensitization and a distinct inflammatory profile [104,105]. Immunologically, LOAD is characterized by predominant Th1 and Th17 activation rather than the Th2-dominant response typical of pediatric forms, resulting in increased production of IFN-γ, IL-17A, and IL-22. These cytokines promote epidermal hyperplasia, keratinocyte dysfunction, and chronic inflammation. Reduced expression of filaggrin, claudins, and other barrier proteins further impairs epidermal integrity [106].

Immunosenescence contributes to dermatitis pathogenesis through functional alterations in T cell populations. Effector T cells become exhausted, in part due to PD-1-mediated inhibitory signaling, while FOXP3^+^ regulatory T cells increase in an attempt to counterbalance systemic low-grade inflammation associated with inflammaging [93]. Senescent T cells sustain chronic inflammation and impair resolution, while age-related structural skin changes—including epidermal thinning, decreased gland density, reduced repair capacity, and lower hydration—facilitate allergen penetration and chronic disease persistence [64].

Allergic contact dermatitis (ACD) is also increasingly common in the elderly and may coexist with LOAD, further complicating diagnosis. Sensitization to metals, fragrances, antiseptics, and topical antibiotics (e.g., neomycin) increases with age due to cumulative exposure and reduced peripheral immune tolerance [107].

Increasing attention has recently been directed toward the contribution of the nervous system and neurogenic inflammation in allergic contact dermatitis. This process is mediated by cutaneous sensory peptidergic nerve fibers and mast cell degranulation via the mast cell-bound MRGPRX2 G protein-coupled receptor, with tryptase playing a predominant role compared with histamine and serotonin [108]. Substance P, released from capsaicin-sensitive unmyelinated sensory nerve fibers, acts primarily through tachykinin neurokinin-1 (NK-1) receptors and is a potent inducer of non-IgE-mediated mast cell degranulation [109]. These mechanisms may contribute to clinical manifestations such as non-histaminergic chronic pruritus and neurodermatitis [55]. Beyond cumulative lifetime exposure to sensitizing haptens—particularly in women—neuroendocrine alterations and vitamin D deficiency may further enhance the expression of neuropeptides and their receptors in keratinocytes [110].

## 6. Food Allergy and Immunosenescence

The global prevalence of food allergies continues to rise, representing an increasing public health concern. Although food allergies are most commonly diagnosed in childhood, recent evidence shows a growing number of cases in adults—and even elderly individuals—suggesting shifts in disease epidemiology [111]. Multiple factors contribute to the development of food allergies in older adults, including immunosenescence and age-related nutritional and environmental changes.

From an immunological standpoint, aging is associated with an imbalance between Th1 and Th2 responses, with a shift toward Th2 dominance, which favors allergic sensitization. This imbalance may be exacerbated by nutritional deficiencies frequently encountered in older adults, such as zinc, iron, and vitamin D deficiencies, which impair immune regulation and further promote Th2-driven responses [112]. Additional contributing mechanisms include dendritic cell dysfunction and reduced activity of effector cells, including mast cells and eosinophils. Aging also alters mucosal immunity, leading to reduced production of secretory IgA, a key defender of the intestinal barrier that prevents allergen translocation [113]. Collectively, these alterations reflect core features of immunosenescence at the gut–immune interface, where impaired antigen handling, reduced immune regulation, and chronic low-grade inflammation converge to disrupt oral tolerance in older adults.

The intestinal epithelial barrier becomes progressively more permeable with age [114], a process worsened by gastric atrophy and by medications such as proton pump inhibitors, which impair protein digestion. This facilitates the passage and presentation of food antigens to the immune system, increasing the likelihood of sensitization [115]. Alterations in the gut microbiota also play a crucial role. Aging and frequent antibiotic exposure reduce microbial diversity [52,53], promote dysbiosis, reduce short-chain fatty acid production, weaken intestinal barrier integrity, and alter Toll-like receptor signaling, allowing greater interaction between luminal antigens and the immune system. This activates local immune responses and triggers the release of inflammatory mediators such as IL-25, IL-33, and thymic stromal lymphopoietin (TSLP), which drive naïve T cells toward a Th2 phenotype [116]. The resulting IgE production and accumulation of mast cells and eosinophils in tissues establish sensitization. Subsequent exposure to the same food allergen may elicit clinical reactions ranging from mild cutaneous symptoms to chronic diarrhea or, in severe cases, life-threatening anaphylaxis [117,118]. At the same time, age-related alterations in gut-associated lymphoid tissue lead to impaired antigen presentation and reduced induction of regulatory T cells, compromising the maintenance of immune tolerance to dietary antigens.

Diagnosing food allergies in older adults is challenging and often delayed due to atypical or nonspecific presentations. Symptoms may be obscured by polypharmacy or overlap with age-related dermatologic, gastrointestinal, or inflammatory disorders [119]. In this context, dysbiosis has been linked to the development of food allergy. The gut microbiota plays a key role in strengthening tight-junction protein expression and maintaining intestinal barrier integrity. Although alterations in both the skin and gut microbiomes have been associated with food allergy, the specific bacterial taxa involved have not yet been clearly identified [120]. Moreover, xerosis, hyperkeratosis, pruritus, increased susceptibility to infection, urticaria, and dermatitis may mimic or mask allergic manifestations. Furthermore, hematologic or immune disorders common in older adults may produce cutaneous findings that resemble allergic reactions, emphasizing the need for careful, multidisciplinary assessment [121].

Taken together, age-related epithelial barrier dysfunction, altered mucosal immunity, microbiota dysbiosis, and inflammaging represent interconnected molecular mechanisms [122] through which immunosenescence contributes to the development, persistence, and atypical presentation of food allergy in the elderly. This immunosenescence-driven environment may also explain the increasing recognition of late-onset food allergy in this age group.

## 7. Hymenoptera Venom Allergy and Aging

Hymenoptera venom allergy (HVA) is an IgE-mediated hypersensitivity disorder triggered by cross-linking of venom-specific IgE antibodies bound to the high-affinity FcεRI receptors on mast cells and basophils. It represents one of the leading causes of anaphylaxis [123,124]. In European population-based studies, the estimated prevalence ranges from 7.5% to 8.9%. Clinical manifestations may occur at any age and vary widely, from mild local reactions to life-threatening systemic responses [125]. For individuals with venom-induced anaphylaxis, venom-specific immunotherapy remains the only disease-modifying treatment, despite limited evidence in the elderly population. 

Age-related comorbidities are important determinants of increased anaphylaxis severity in older adults [126]. Cardiovascular disease may impair compensatory responses during anaphylaxis [127]. In addition, commonly prescribed medications, particularly β-blockers and ACE inhibitors, can interfere with physiological compensatory mechanisms such as vasoconstriction and bronchodilation, and may reduce the efficacy of epinephrine, thereby increasing the risk of severe or fatal reactions.

At the cellular level, mast cell immunosenescence contributes to enhanced systemic and cardiovascular reactivity, whereas bronchial involvement may be attenuated due to a reduced number or impaired function of pulmonary mast cells [128]. This may explain the predominantly cardiovascular, rather than respiratory, clinical presentation described in older patients by Pawłowicz et al. (2024) [129]. Moreover, age-related deterioration in mast cell function—including impaired signal transduction, activation, survival, and mediator release—has been proposed as a potential risk factor for more severe HVA in the elderly. Therefore, advancing age should be recognized as a factor that may increase the severity of HVA reactions. Elevated baseline serum tryptase levels, particularly when combined with aging, may further augment this risk [130,131].

## 8. Drug Allergy in the Elderly

Drug allergy (DA) is an adverse drug reaction resulting from a specific immunological response to a medication. It is generally unpredictable and occurs only in susceptible individuals. According to the classification proposed by Rawlins and Thompson, adverse drug reactions (ADRs) are categorized as type A (augmented), type B (bizarre), type C (chronic), type D (delayed), type E (end-of-use), and type F (failure). Immunologically mediated reactions may involve humoral mechanisms (IgE or IgG antibodies) or T lymphocyte-mediated mechanisms [132].

While the prevalence and risk factors associated with ADRs in the general adult population are well documented, considerably less is known about ADRs, and particularly immunologically mediated DAs, in older adults. Several age-related factors increase susceptibility to ADRs, including frailty, multimorbidity, cognitive impairment, and polypharmacy, which involves potentially interacting prescription and over-the-counter medications. Drug-related hospitalizations account for 2.4–6.5% of all medical admissions in the general population, with substantially higher rates observed among older patients [133].

Epidemiological studies indicate that drug classes most frequently associated with ADRs in the elderly include diuretics, warfarin, nonsteroidal anti-inflammatory drugs, selective serotonin reuptake inhibitors, β-blockers, angiotensin-converting enzyme inhibitors, cancer chemotherapeutic agents, and glucocorticoids [134]. Data from the Italian Group for Pharmacovigilance in the Elderly suggest that age itself is not an independent risk factor but is associated with significant alterations in pharmacokinetics and pharmacodynamics that predispose older patients to adverse reactions. Polypharmacy, age-related metabolic and physiological changes, and drug–drug interactions represent the main contributors to ADR risk in this population.

To date, no robust epidemiological studies have specifically defined the prevalence of true drug allergy in the elderly, and most ADRs reported in this age group are not genuine immunologically mediated allergies. Available evidence suggests that DA in older adults has an estimated prevalence of 0.6–2.1%, yet it may account for up to 10% of fatal adverse reactions [135].

Immunosenescence, due to the reduced immune hyperreactivity, may also contribute to the lower incidence of vaccine-related hypersensitivity reactions in older adults compared with younger individuals [136,137]. This trend was observed during the COVID-19 vaccination campaign, where pharmacovigilance reports from AIFA and EMA demonstrated a clear predominance of systemic adverse reactions in younger adults, consistent with the less hyperergic immune profile typical of aging [138,139].

## 9. Diagnostic and Therapeutic Implications of Aging

Immunosenescence profoundly influences both diagnostic accuracy and therapeutic decision-making in allergic diseases, requiring a tailored and age-aware approach. Immunosenescence may reduce the accuracy of skin testing, including patch tests and provocation tests, due to diminished cutaneous reactivity. In angioedema, acquired C1-INH deficiency should be excluded through functional and immunochemical assessment, particularly in refractory cases [140,141,142]. Serum tryptase, useful in diagnosing anaphylaxis and mastocytosis, may show age-related increases and should therefore be interpreted with caution [143].

Although biologic therapies have undoubtedly expanded treatment options, their use in older adults exposes a central dilemma between demonstrated efficacy and the limited availability of age-specific evidence, particularly in the context of immune aging, multimorbidity, and long-term safety. Immunosenescence may influence both therapeutic responsiveness and susceptibility to adverse events, making extrapolation from younger populations potentially misleading. Nevertheless, omalizumab (anti-IgE) is effective in refractory chronic urticaria [144,145], and dupilumab (IL-4/IL-13 blockade) has demonstrated excellent outcomes in atopic dermatitis, including in patients over 65 years [146,147].

Among other therapeutic options, monoclonal antibodies targeting IL-5 or the IL-5 receptor can respectively reduce or deplete eosinophils in respiratory allergic diseases [148]. Within a hypothetical hierarchy of targeted treatments, anti-thymic stromal lymphopoietin (TSLP) monoclonal antibody, directed against an epithelial alarmin, occupies the highest level of upstream intervention [149]. Allergen-specific immunotherapy remains a cornerstone of allergy management from childhood onward, functioning as a disease-modifying treatment capable of inducing long-lasting immune tolerance through the expansion of regulatory T cells and the increased production of IL-10 [150]. In the field of atopic dermatitis, inhibition of the JAK/STAT cascade, and thus suppression of downstream signaling driven by pro-inflammatory cytokines, can be achieved with small molecules such as upadacitinib and baricitinib [151].

Moreover, therapeutic choices in older adults should not be driven solely by disease severity but by a comprehensive assessment of biological age. Therapeutic decisions must be individualized, considering renal function, cardiovascular disease, polypharmacy, nutritional status, frailty, and potential side effects [152,153]. Particular attention should be given to cardiovascular and thrombotic risk, the increased susceptibility to infections, especially herpes zoster, and the underlying condition of immunosenescence itself (Table 2), despite the limited number of available studies, highlighting the urgent need for dedicated studies in geriatric allergic populations.

Ultimately, from a therapeutic perspective, the increase in neurogenic inflammation associated with neuro-immune aging offers promising avenues for future treatments, such as small-molecule MRGPRX2 antagonists, particularly for chronic dermatoses [156]. In addition, pruritogenic stimuli in cutaneous disorders can activate cannabinoid receptors 1 and 2 on sensory neurons, suggesting that non-THC cannabinoid-based topical treatments may represent an interesting modulatory option, especially in refractory non-histaminergic prurigo [157,158].

## 10. Allergy and Gender in Immunosenescence

Genetic, hormonal, and chromosomal differences between males and females exert a significant influence on immune function [159,160]. The X chromosome, which carries numerous genes involved in immune regulation, plays a central role. Women, who possess two X chromosomes, show a higher predisposition to autoimmune and allergic diseases [161], whereas men, having only one X chromosome, appear comparatively protected. Key immunological genes located on the X chromosome, including TLR7 and TLR8, regulate interactions with B cells and the gut microbiota, thereby shaping immune responses. The presence of two X chromosomes in women results in greater expression of these genes, contributing to increased susceptibility to allergic and autoimmune conditions [162]. In contrast, the single X chromosome in men is associated with a lower risk of developing such disorders [163].

Sex hormones also play an essential immunomodulatory role. Androgens suppress immune activation by reducing the activity of type 2 innate lymphoid cells (ILC2), which are central to Th2-driven inflammatory pathways characteristic of allergic disease [164]. This androgen-mediated inhibition of ILC2 responses provides relative protection against allergic inflammation in males, helping explain the lower prevalence of asthma in post-pubertal males compared with females, in whom this protective mechanism is less prominent [165]. Conversely, estrogens tend to enhance immune responses during infections, following vaccination, and in autoimmune conditions.

Evidence examining how aging modifies these sex-related immunological differences remains limited. However, epidemiological data suggest that women progressively lose their immunological advantage after menopause, likely due to declining estrogen levels and associated immune remodeling [166].

An exception is hymenoptera venom allergy, which in older adults also appears to remain skewed toward men, likely due to gender-related factors, particularly greater exposure, both occupational and outdoor, including past exposure [129]. Similarly, mastocytosis, a genetically driven disease characterized by KIT mutations that lead to aberrant mast cell degranulation and elevated baseline serum tryptase levels, is more frequent in men, including in older age, particularly in systemic mastocytosis with associated clonal hematological non-mast cell lineage and advanced or late-diagnosed forms [167].

## 11. Discussion

**Figure 2:**
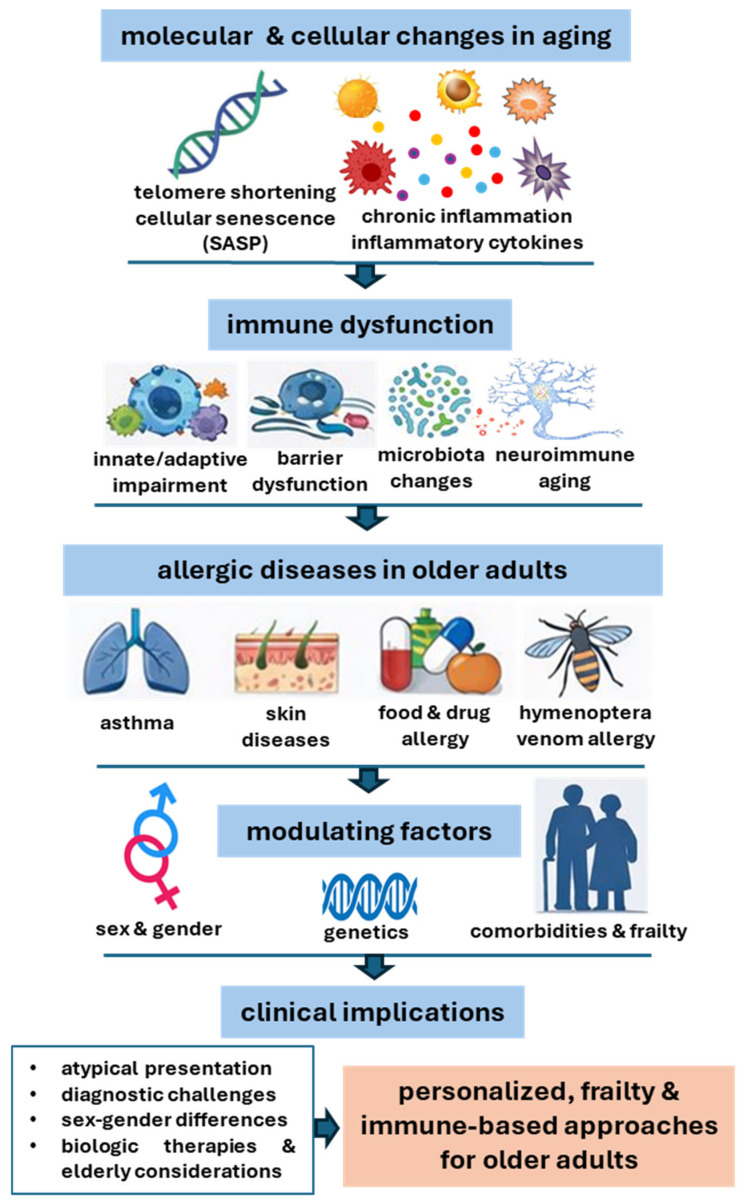
Allergic diseases in aging: immunosenescence, inflammaging, and sex differences. Age-related molecular and cellular changes, including immunosenescence and inflammaging, lead to immune dysfunction in older adults. These processes, modulated by genetic and sex- and gender-related factors, influence the pathophysiology, clinical expression, and management of major allergic diseases. Clinical implications include diagnostic challenges, sex–gender differentiation, elderly-specific considerations for biologic therapies, and the need for personalized approaches based on frailty and immune profiling. SASP: senescence-associated secretory phenotype.

This review synthesizes current knowledge on the impact of immunosenescence on allergic diseases and aims to identify future directions for research and clinical practice. As the geriatric population continues to grow, it becomes increasingly important to understand how age-related immunological changes influence diagnosis, clinical presentation, and therapeutic response in older adults. By integrating cellular and molecular evidence with clinical observations, this review examines asthma, urticaria, dermatitis, food allergy, drug allergy, and hymenoptera venom allergy, offering translational insight relevant to both clinicians and researchers (Figure 2).

In asthma, pulmonary aging is associated with low-grade chronic inflammation (inflammaging) and mixed Th2/Th17 immunity, which complicate diagnostic assessment and render responses to biologic therapies less predictable. However, available data suggest a favorable safety profile, even in patients over 70 years of age, at least for the biologics most widely used in this age group to date. With regard to respiratory allergic diseases, there remains a need for improved education on inhaler and device use in the elderly, even in milder forms, as well as for concomitant management of underlying rhinopathy, as previously discussed.

Conversely, systemic corticosteroid use increases with advancing age and disease severity, leading to predictable adverse effects and negative interactions with comorbidities typical of this age group. Therefore, in severe or poorly controlled cases, priority should be given to biologic therapies, such as omalizumab [168]. Dupilumab also appears to show promising results across multiple type 2-driven diseases in elderly patients, whereas for therapies targeting eosinophils, it may be advisable to avoid strongly depleting mechanisms, given the physiological role of eosinophils [169] in the context of immunosenescence. Immunosenescence itself may also contribute to the reduced effectiveness of immunomodulatory strategies, such as allergen-specific immunotherapy, despite its well-established efficacy in other age groups. In cutaneous diseases, the frailty and multimorbidity characteristic of older adults require particular caution when using innovative therapies within the class of small molecules, such as Janus kinase (JAK) inhibitors.

In this context, the need for more sensitive diagnostic tools to better characterize allergic pathophysiology in relation to cellular senescence may be addressed through the analysis of potential biomarkers such as CD28^−^CD57^+^ T cells, IL-2, and IL-6. These markers may allow for a more precise qualitative and quantitative assessment of immunocompetence in older adults. When appropriately integrated, such data could provide valuable insights for tailoring the immunomodulatory or immunosuppressive effects of advanced immunologic therapies in this age group, both for treatment selection and therapeutic monitoring, while aiming to avoid further increasing the already elevated susceptibility to infections and malignancies associated with aging.

In parallel, assessment of substance P may represent a hallmark of neuro-immune aging, reflecting the amplification of neurogenic inflammatory responses and functional alterations in key effector cells, such as mast cells, in elderly individuals. Given the potential activation of non-conventional molecular and cellular pathways, it is essential to distinguish pseudo-allergic manifestations from truly IgE-mediated allergic reactions. Etiologically, this distinction helps explain the limited responsiveness to antihistamines observed in clinical presentations that are not classically histaminergic. In this regard, we have briefly outlined some promising future perspectives, including modulation of the mast cell-associated MRGPRX2 receptor and the endocannabinoid system as potential therapeutic strategies for refractory chronic pruritus and neurodermatitis.

In addition, a shift away from a typical Th2-driven pattern toward T2-low or non–T2 endotypes may support the preferential investigation of tezepelumab among available biologic therapies in this age group, particularly when a therapeutic step-up beyond conventional treatments is clinically required [149,170].

Food allergies also appear to be increasing in older adults, supported by mechanisms such as intestinal barrier dysfunction, dysbiosis, and impaired mucosal immunity. Although immune remodeling during aging can promote a chronic Th1-skewed pro-inflammatory milieu, elderly individuals may nonetheless exhibit increased serum IgE levels, which can correlate with Th2-type responses and the development of food allergy [125]. However, diagnosis is frequently delayed because symptoms may be atypical or overlap with non-allergic dermatologic and gastrointestinal conditions commonly observed in older age.

Allergic skin diseases, including urticaria, angioedema, and dermatitis, display characteristic patterns in older adults, such as subtle or atypical cutaneous manifestations, a higher prevalence of autoimmune endotypes, and increased involvement of bradykinin-mediated pathways. In this context, acquired C1 esterase inhibitor deficiency secondary to lymphoproliferative disorders emerges as a key differential diagnosis. In dermatitis, accumulating evidence indicates marked epidermal barrier dysfunction and a shift toward Th1/Th17-driven inflammatory responses.

In hymenoptera venom allergy, advancing age correlates with an increased risk of cardiovascular manifestations and more severe anaphylaxis, likely reflecting reduced mast cell reserve, impaired compensatory mechanisms, and the high prevalence of cardiovascular comorbidities. Venom immunotherapy remains effective, although evidence in individuals older than 65 years is still limited. Moreover, while overall exposure to hymenoptera stings may be lower in older adults, particularly with respect to occupational exposure, severe or potentially fatal reactions cannot be excluded, even following accidental stings.

Drug-related reactions represent a major clinical challenge, not only because of their frequency but also due to the difficulty of distinguishing true immune-mediated drug allergy from non-immunologic adverse drug reactions in the context of polypharmacy, cognitive decline, multimorbidity, and altered pharmacokinetics and pharmacodynamics. These variables should inform future prescribing recommendations and risk–benefit analyses, including the personalization of therapeutic pathways when choosing between conventional and advanced anti-allergic treatments, whether administered as monotherapy or in combination. Interactions with long-term background medications commonly used in this age group and those most frequently responsible for adverse reactions—which differ from those observed in younger adults—should also be investigated more systematically [171]. This approach would support the development of age-specific guidance for the management and appropriate use of these therapies, without unnecessarily restricting their availability in older patients. In selected cases, optimization strategies may also include dose adjustments, particularly dose reduction or modified administration intervals, beyond those recommended for the general population.

A recurring theme across allergic conditions is the role of sex-related differences, which remain underrecognized. Enhanced immune activation in women, linked to X-chromosome-encoded immune genes and lower androgen levels, likely contributes to the higher prevalence of allergic diseases observed in elderly females.

Finally, it is noteworthy that osteoporosis—a condition typically associated with advanced age and predominantly affecting women—has been linked to increased levels of IL-31, a cytokine recognized as a marker of allergic inflammation. This observation supports the hypothesis of a broader pathogenic loop in which medication use, dietary restrictions, chronic low-grade inflammation, and mediators such as histamine and tryptase exert systemic effects on organs not primarily involved in allergic disease, ultimately influencing bone health. These mechanisms may be further amplified by underlying conditions such as hyper-IgE syndromes, mastocytosis, and hypereosinophilic syndromes, particularly in elderly patients [172,173].

This review, however, has certain limitations, primarily the paucity of studies specifically focused on older adults, which restricts the ability to accurately define prevalence, phenotypes, and immunopathological mechanisms in this age group. A major limitation in the current literature on allergic diseases is the persistent lack of studies specifically designed for older adults. Elderly patients are frequently underrepresented or excluded from clinical trials, and age-stratified analyses are often unavailable. Moreover, much of the available evidence is derived from heterogeneous adult cohorts without age stratification, introducing potential interpretative bias. Even in very recent real-life cohorts of patients treated with omalizumab or dupilumab, respectively for chronic spontaneous urticaria and for Th2-mediated inflammatory respiratory involvement, or atopic dermatitis, and prurigo nodularis, the mean ± standard deviation and the median age are well below 65 years [174,175].

As a result, diagnostic criteria, biomarker interpretation, and therapeutic efficacy are largely extrapolated from younger populations, despite profound age-related differences in immune function, comorbidity burden, and drug responsiveness. This gap significantly limits the development of evidence-based, age-appropriate management strategies and underscores the urgent need for dedicated epidemiological studies and clinical trials specifically targeting the geriatric population.

Future research should include longitudinal studies evaluating immunological aging and its relationship to allergic disease onset, as well as dedicated clinical trials assessing the efficacy and safety of biologics in elderly populations, including therapies such as omalizumab for asthma, urticaria, and nasal polyposis.

Future perspectives should promote diagnostic and therapeutic approaches that incorporate frailty, comorbidities, functional status, and individualized immune profiling. A deeper understanding of the molecular mechanisms of immunosenescence may contribute to the development of biomarkers and targeted therapies, considering the canonical and non-canonical allergic pathways and the contribution of neuroinflammation and neuro-immune aging, ultimately improving personalized allergy care in older adults.

## 12. Conclusions and Future Perspectives

Immunosenescence represents both a challenge and an opportunity for understanding and managing allergic diseases in the elderly. Age-related immune alterations influence clinical presentation, diagnostic accuracy, and therapeutic responsiveness, underscoring the need for a multidisciplinary and individualized approach. Current evidence suggests that age should be regarded not merely as a demographic descriptor, but as a relevant immunological variable capable of modifying disease mechanisms and outcomes, including neuro-immune aging and responses to biologic therapies. To improve care, future efforts should focus on developing clinical studies specifically designed for elderly populations, refining diagnostic strategies that consider comorbidities, frailty, and atypical test responses, and implementing personalized therapeutic approaches, as well as the use of biologics and small molecules, either approved or currently under investigation, guided by a careful assessment of risks and benefits. Only through this integrated perspective will it be possible to ensure equitable, evidence-based, and effective management of allergic diseases in an increasingly long-lived society.

## Figures and Tables

**Table 1:** Key age-related molecular pathways contributing to immunosenescence, inflammaging, and allergic diseases.

Pathway	Key Molecules/Cells	Functional Impact	Clinical Implications
T cell senescence	CD28^−^CD57^+^ T cells; IL-2; IL-6;	Reduced IL-2; altered Th1/Th2/Th17 balance.	Chronic inflammation; atypical allergic manifestations; reduced tolerance [21,23].
B cell aging	Exhausted memory B cells	Reduced class switching; impaired affinity maturation	Lower IgE/IgG antibody affinity; altered allergic reactivity [24,26].
Mast cell remodeling	FcεRI; ROS	Altered degranulation; ↑ basal inflammatory mediators.	Atypical urticaria; chronic eczema; neurogenic inflammation [27,28].
Eosinophil decline	IL-5R; GM-CSF	Reduced chemotaxis; impaired survival.	Modified asthma phenotype; variable biologic response [17,29].
SASP	IL-6; IL-8; IL-1β; TNF-α; MMPs; alarmins	Chronic sterile inflammation; barrier dysfunction.	Persistent allergic inflammation; inflammaging [35,36,50]
Mitochondrial dysfunction	ROS; NLRP3 inflammasome	↑ IL-1β/IL-18; impaired repair; oxidative stress.	Inflammatory exacerbations; tissue fragility [32,33,34,37,51].
Microbiome dysbiosis	SCFAs; TLR ligands	Reduced epithelial integrity; ↑ allergen penetration.	Altered barrier function and immune tolerance; allergen penetration and sensitization; chronic inflammation [31,38,39,52,53].
Neuro-immune aging	Substance P; CGRP	Increased neurogenic inflammation.	Chronic pruritus; neurodermatitis; non-IgE-mediated hypersensitivity [40,41,42,54,55].

Aging remodels both innate and adaptive immune responses through T cell senescence, impaired B cell function, mast cell reprogramming, eosinophil decline, SASP-driven inflammation, mitochondrial dysfunction, microbiome dysbiosis, and neuro-immune alterations. These mechanisms collectively weaken immune tolerance, enhance tissue inflammation, and influence disease severity, phenotype, and therapeutic responsiveness in older allergic patients. SASP: senescence-associated secretory phenotype; ROS: reactive oxygen species; IL: interleukin; TNF-α: tumor necrosis factor-alpha; CD: cluster of differentiation; GM-CSF: granulocyte–macrophage colony-stimulating factor; FcεRI: high-affinity IgE receptor; MMPs: matrix metalloproteinases; NLRP3: NACHT, LRR and PYD domain-containing protein 3 inflammasome; SCFA: short-chain fatty acids; TLR: Toll-like receptor; Th: T-helper cell; CGRP: calcitonin gene-related peptide.

**Table 2:** Overview of targeted therapies used in older adults with allergic and immunosenescence-related conditions.

Drug Class/Target	Mechanism of Action	Key Points in the Elderly and Safety Considerations
Anti-IgE (Omalizumab)	Neutralizes free IgE; downregulates FcεRI	Effective in elderly asthma and CSU. Monitor for cardiovascular comorbidities; rare anaphylaxis [144,145].
Anti-IL-5 (Mepolizumab)	Reduces eosinophil survival	Good responses despite age-related eosinophil decline; monitor infections (herpes zoster) [148,154].
Anti-IL-5R (Benralizumab)	Depletes eosinophils via ADCC	Useful in severe eosinophilic asthma. Caution for immunosenescence-related fragility [148,155].
Anti-IL-4/IL-13 (Dupilumab)	Blocks IL-4Rα; inhibits T2 pathways	Effective for AD, CRSwNP, asthma in older adults; conjunctivitis; arthralgias; monitoring in multimorbidity [146,147].
Anti-TSLP (Tezepelumab)	Inhibits epithelial alarmin TSLP	Suitable for T2-low and mixed phenotypes; monitor infections; unknown long-term effects in older adults [149].
JAK inhibitors (upadacitinib, baricitinib)	Blocks JAK-STAT signaling	Efficacy in AD; ↑ risk of thrombosis, herpes zoster; use with caution [151].
Allergen immunotherapy	Induces tolerance via Treg/IL-10	Less effective due to immunosenescence; monitor for systemic reactions [150].

The table summarizes the principal biological targets involved, the mechanisms of action of anti-IgE, anti-IL-5/IL-5Rα, anti-IL-4Rα, and anti-TSLP agents and small molecules JAK inhibitors, specific aspects of allergen immunotherapy in older adults; moreover, potential effects of immunosenescence on treatment response and safety considerations related to comorbidities and polypharmacy common in the elderly are highlighted. IgE: immunoglobulin E; IL-5: interleukin-5; IL-4Rα: interleukin-4 receptor alpha; TSLP: thymic stromal lymphopoietin; ADCC: antibody-dependent cell-mediated cytotoxicity; JAK: Janus kinase; CSU: chronic spontaneous urticaria; AD: atopic dermatitis; CRSwNP: chronic Rhinosinusitis with nasal polyps.

## Data Availability

No new data were created or analyzed in this study. Data sharing is not applicable to this article.
